# A Distinct Pool of Na_v_1.5 Channels at the Lateral Membrane of Murine Ventricular Cardiomyocytes

**DOI:** 10.3389/fphys.2019.00834

**Published:** 2019-07-03

**Authors:** Jean-Sébastien Rougier, Maria C. Essers, Ludovic Gillet, Sabrina Guichard, Stephan Sonntag, Doron Shmerling, Hugues Abriel

**Affiliations:** ^1^Institute of Biochemistry and Molecular Medicine, University of Bern, Bern, Switzerland; ^2^Pain Center, Department of Anesthesiology, Lausanne University Hospital, Lausanne, Switzerland; ^3^Department of Fundamental Neurosciences, University of Lausanne, Lausanne, Switzerland; ^4^PolyGene AG, Rümlang, Switzerland

**Keywords:** voltage-gated sodium channel, lateral membrane of cardiomyocytes, α1-syntrophin, dystrophin, electrophysiology

## Abstract

**Background:** In cardiac ventricular muscle cells, the presence of voltage-gated sodium channels Na_v_1.5 at the lateral membrane depends in part on the interaction between the dystrophin–syntrophin complex and the Na_v_1.5 C-terminal PDZ-domain-binding sequence Ser-Ile-Val (SIV motif). α1-Syntrophin, a PDZ-domain adaptor protein, mediates the interaction between Na_v_1.5 and dystrophin at the lateral membrane of cardiac cells. Using the cell-attached patch-clamp approach on cardiomyocytes expressing Na_v_1.5 in which the SIV motif is deleted (ΔSIV), sodium current (I_Na_) recordings from the lateral membrane revealed a SIV-motif-independent I_Na_. Since immunostaining has suggested that Na_v_1.5 is expressed in transverse (T-) tubules, this remaining I_Na_ might be carried by channels in the T-tubules. Of note, a recent study using heterologous expression systems showed that α1-syntrophin also interacts with the Na_v_1.5 N-terminus, which may explain the SIV-motif independent I_Na_ at the lateral membrane of cardiomyocytes.

**Aim:** To address the role of α1-syntrophin in regulating the I_Na_ at the lateral membrane of cardiac cells.

**Methods and Results:** Patch-clamp experiments in cell-attached configuration were performed on the lateral membranes of wild-type, α1-syntrophin knockdown, and ΔSIV ventricular mouse cardiomyocytes. Compared to wild-type, a reduction of the lateral I_Na_ was observed in myocytes from α1-syntrophin knockdown hearts. Similar to ΔSIV myocytes, a remaining I_Na_ was still recorded. In addition, cell-attached I_Na_ recordings from lateral membrane did not differ significantly between non-detubulated and detubulated ΔSIV cardiomyocytes. Lastly, we obtained evidence suggesting that cell-attached patch-clamp experiments on the lateral membrane cannot record currents carried by channels in T-tubules such as calcium channels.

**Conclusion:** Altogether, these results suggest the presence of a sub-pool of sodium channels at the lateral membrane of cardiomyocytes that is independent of α1-syntrophin and the PDZ-binding motif of Na_v_1.5, located in membrane domains outside of T-tubules. The question of a T-tubular pool of Na_v_1.5 channels, however, remains open.

## Introduction

The sarcolemma of rod-shaped adult ventricular cardiomyocytes can be divided into two principal regions. Firstly, the intercalated disc (ID) allows the electrical and physical coupling between adjacent cardiac cells *via* ID-ID connections. Secondly, the lateral membrane, including transverse (T-) tubules, contains focal adhesions crucial for the interaction between cardiomyocytes and the extracellular matrix (Kaprielian and Severs, 2000; Noorman et al., 2009; Vermij et al., 2017). The voltage-gated sodium channel Na_v_1.5 is expressed in these membrane domains. Na_v_1.5 is the major isoform of the Na_v_1.*x* family in cardiomyocytes (Chevalier et al., 2018). These channels are involved in the generation and propagation of the cardiac action potential (AP) (Gavillet et al., 2006; Petitprez et al., 2011). A few years ago, we and other groups investigating Na_v_1.5 started to unveil the complex localization of this channel in cardiac cells. We showed that at least two pools of Na_v_1.5 channels are present in murine ventricular cardiomyocytes based on their expression pattern and interaction with cytoplasmic proteins (Abriel et al., 2015). The first pool is located at the ID and plays a crucial role in the propagation of the electrical impulse. Based on results from heterologous expression systems and transfected cardiac cells, the regulation of this pool has been proposed to depend on SAP97 (synapse-associated protein 97), although *in vivo* experiments using cardiac-specific SAP97 knockdown mice did not support such observations (Petitprez et al., 2011; Milstein et al., 2012; Gillet et al., 2015). Recently, Makara et al. (2014) have convincingly shown the crucial role of ankyrin-G in the regulation and targeting of Na_v_1.5 channel at the ID *in vivo*. The second pool of Na_v_1.5 channels has been described outside the ID domain comprising the lateral membrane and the T-tubular system (Gavillet et al., 2006; Albesa et al., 2011; Shy et al., 2014). Contrary to what was thought commonly, the presence of Na_v_1.5 channels at the lateral membrane seems to be important for cardiac conduction (Baba et al., 2005; Shy et al., 2014). At the lateral membrane, the presence of Na_v_1.5 channels mainly depends on the protein dystrophin and the PDZ-binding motif of Na_v_1.5 comprising its last three C-terminal amino acids Ser, Ile, and Val (SIV). The stability of Na_v_1.5 at the lateral membrane has been shown to depend on the interaction between the dystrophin–syntrophin complex and the SIV motif (Gavillet et al., 2006; Shy et al., 2014). In this interaction, syntrophin, containing a PDZ domain, act as an adaptor protein between the SIV motif of Na_v_1.5 and dystrophin. Depending on the species, dystrophin can interact with different isoforms of syntrophin (Iwata et al., 2005). The syntrophin family comprises five isoforms of ∼60 kD each: α1, ß1, ß2, γ1, and γ2. α1-Syntrophin is abundantly expressed in mouse heart (Yang et al., 1995). The γ-isoforms are mainly expressed in the brain and skeletal muscle. α- and ß- isoforms are expressed in many tissues including the heart. In hamster, rat, and canine ventricular cardiomyocytes, dystrophin has been shown to interact preferentially with the α1-, β1-, and β2-isoforms of syntrophin (Iwata et al., 2005). In human and rabbit ventricles, mainly the α1-isoform interacts with dystrophin (Iwata et al., 2005). Using the cell-attached patch-clamp recording method on ventricular cardiomyocytes from genetically modified mice expressing Na_v_1.5 lacking the SIV motif (ΔSIV mouse model), a ∼60% reduction, but not an abolition, of I_Na_ has been observed at the lateral membrane (Shy et al., 2014). In addition, the biophysical properties of the remaining I_Na_ at the lateral membrane of ΔSIV myocytes were similar to the wild-type cells from littermate mice (Shy et al., 2014). Shy et al. (2014) proposed that this remaining current originates from Na_v_1.5 channels at the T-tubules. However, the presence of Na_v_1.5 in T-tubular system is still under debate. Although immunofluorescence staining for Na_v_1.5 has shown a T-tubular-like pattern in ventricular cardiomyocytes, other experiments suggest that other Na_v_1.*x* isoforms might be the main isoforms in this structure (Brette and Orchard, 2006; Westenbroek et al., 2013; Shy et al., 2014). Interestingly, Matamoros and colleagues, have shown in heterologous expression systems that α1-syntrophin cannot only interact with the C-terminal SIV motif but also with the sequence comprising three N-terminal amino acids Ser, Leu, Ala (SLA) of Na_v_1.5 channels (Matamoros et al., 2016). Based on this observation, the remaining I_Na_ observed on the lateral membrane of ΔSIV mice could also depend on α1-syntrophin interacting with Na_v_1.5 through the N-terminal SLA motif.

Taken together, one might explain the remaining I_Na_ outside the ID in ΔSIV ventricular cardiomyocytes as follows: (1) Na_v_1.5 channels also interact with the dystrophin–syntrophin complex *via* another mechanism; and/or (2) a subset of Na_v_1.5 channels at the lateral membrane are independent of the dystrophin–syntrophin complex; and/or (3) Na_v_1.5 channels are present inside the T-tubular system. In this work, we aimed to test these hypotheses.

## Materials and Methods

All experiments involving animals were performed according to the Swiss Federal Animal Protection Law and had been approved by the Cantonal Veterinary Administration, Bern. This investigation conforms to the Guide for the Care and Use of Laboratory Animals, published by the US National Institutes of Health (NIH publication no. 85–23, revised 1996).

### Generation of Constitutive ΔSIV (*Scn5a*-p.S2017Stop) Knockin Mice

Generation of mice containing the ΔSIV knockin deletion was accomplished through the Cre/lox technique and homologous recombination. Briefly, the serine 2017 residue was mutated to a stop codon and a silent *EcoRV* site was introduced within the 3′ UTR to allow genotyping. Heterozygous *Scn5a*-p.S2017STOP mice were crossbred to obtain homozygous *Scn5a*-p.S2017STOP mice. Wild-type littermates served as control mice (Shy et al., 2014).

### Generation of Constitutive Cardiac-Specific α1-Syntrophin Knockdown Mice

To study the function of α1-syntrophin (Sntα1), a conditional mouse model was generated that allows tissue or time-point specific inactivation of the *Snta1* gene by the use of the Cre-/loxP-system. The design of the mouse model is based on a modified *Snta1* locus with two loxP sites flanking part of *Snta1* exon1. One loxP site is inserted in the 5′ UTR in exon1, whereas the second loxP site is located in intron 1. Upon breeding with a heart-specific Cre-transgenic mouse line (i.e., MHC-Cre), the coding region on exon 1 of *Snta1* is deleted, restricted to the cardiac muscle that is studied. For the introduction of loxP sites into the *Snta1* locus, a targeting vector (pSnta1 TV) was generated that contained homology regions of 2.2 kb (short homology region) and 5.6 kb (long homology region). The 3′ loxP site was inserted together with an FRT-flanked neomycin cassette into a KpnI restriction site 269 bp downstream of *Snta1* exon 1. The 5′ loxP site was inserted 50 bp downstream of the start of exon 1 and 78 bp upstream of the ATG start codon in the 5′ untranslated region (5′ UTR). In total a region of 639 bp was flanked by loxP sites, containing the coding part of *Snta1* exon 1. After transfection of 20 mg of the targeting vector into 10^7^ 129Ola derived embryonic stem cells and selection with 200 mg/ml G418 for 10 days, 100 clones were isolated and screened for insertion into the *Snta1* locus via Southern blot analysis using PstI digested genomic DNA and a 5′ external probe (Supplementary Figure S1A). Eight positive clones were identified and further analyzed by Southern blot using PstI digested genomic DNA and a 3′ external probe (Supplementary Figure S1B). Two clones with a correct karyotype were injected into C57BL/6 derived blastocysts. Male mice with a high degree of coat color chimerism were mated to Flp-deleter mice (C57Bl/6 background) to generate heterozygous *Snta1*-flox mice. For genotyping, two primers flanking the 5′ loxP site were used (Supplementary Figure S1A). Primers E072.32 (5′-ACGAGCTACGGTCCAATC-3′) and E072.33 (5′-ATCTTCGCCTCGAAGTCC-3′) detect a 391 bp fragment in the wild-type allele, whereas the insertion of the loxP site in the *Snta1*-flox allele results in a slightly larger fragment of 437 bp.

### Cell Line Preparations

Human embryonic kidney (HEK-293) cells were cultured in DMEM (Gibco, Basel, Switzerland) supplemented with 10% FBS, 0.5% penicillin, and streptomycin (10,000 U/mL) at 37°C in a 5% CO_2_ incubator.

### Transfections

For electrophysiological studies, T25 cm^2^ flasks of HEK-293 cells were transiently co-transfected using Jet-Pei^®^ reagent (Polyplus-transfection SA, Illkirch, France) with 10 μg of human Na_v_1.4 cDNA. All transfections included 0.3 μg of cDNA encoding CD8. For patch clamp experiments, anti-CD8 beads (Dynal^®^, Oslo, Norway) were used. Only cells decorated with anti-CD8 beads were analyzed.

### Isolation of Mouse Ventricular Myocytes

Single cardiomyocytes were isolated according to a modified procedure of established enzymatic methods (Gavillet et al., 2006). Briefly, mice were euthanized by cervical dislocation. Hearts were rapidly excised, cannulated and mounted on a Langendorff column for retrograde perfusion at 37°C. Hearts were rinsed free of blood with a nominally Ca^2+^-free solution containing (in mM): 135 NaCl, 4 KCl, 1.2 MgCl_2_, 1.2 NaH_2_PO_4_, 10 HEPES, 11 glucose, pH 7.4 (NaOH adjusted), and subsequently digested by a solution supplemented with 50 μM Ca^2+^ and collagenase type II (1 mg/mL, Worthington, Allschwil, Switzerland) for 15 min. Following digestion, the atria were removed and the ventricles transferred to nominally Ca^2+^-free solution, where they were minced into small pieces. Single cardiac myocytes were liberated by gentle trituration of the digested ventricular tissue and filtered through a 100 μm nylon mesh. Ventricular mouse cardiomyocytes were used after an extracellular calcium increase procedure to avoid calcium overload when applying extracellular solutions in electrophysiology assays.

### Detubulation

Isolated cardiomyocytes were detubulated *via* osmotic shock using formamide solution (Brette et al., 2002). Briefly, cardiac cells were suspended in 2 mL of 1.5 M formamide solution for 15 min at room temperature (RT) then washed twice with an extracellular solution before use. For morphological experiments, 1 μM di-8 ANEPPS, diluted in dimethyl sulfoxide containing pluronic F-127, was applied to living cardiomyocytes for 15 min, and imaged on a confocal microscope (LSM710, Zeiss, Germany).

### Whole-Cell Electrophysiology

Cardiac APs and ionic currents [I_Na_, calcium current (I_Ca_), transient outward potassium current (I_to_), and the inward rectifier potassium current (I_K1_)] were recorded in the whole-cell configuration at RT (22–23°C) using a VE-2 amplifier (Alembic Instruments, United States). Borosilicate glass pipettes were pulled to a series resistance of ∼2 MΩ. pClamp software, version 8 (Axon Instruments, Union City, CA, United States) was used for recordings. Data were analyzed using pClamp software, version 8 (Axon Instruments) and OriginPro, version 7.5 (OriginLab Corporation, Northampton, MA, United States). Current densities (pA/pF) were calculated by dividing the peak current by the cell capacitance. For sodium currents, current–voltage relationship curves (I–V) were fitted with the equation, *y* = g^∗^(V_m_-V_rev_)/((1+exp[(V_m_-V_1/2_)/K])). To determine the voltage dependence of steady-state activation and inactivation curves, the data from individual cells were fitted with the Boltzmann equation, *y* = 1/(1+exp[(V_m_-V_1/2_)/K]). Y is the normalized current or conductance, V_m_ is the membrane potential, V_1/2_ is the voltage at which half of the channels are activated or inactivated, K is the slope factor, and V_rev_ the reversal potential.

For cardiac AP recordings, cardiomyocytes were bathed in a solution containing (in mM) NaCl 140, KCl 5.4, CaCl_2_ 1.8, MgCl_2_ 1.2, HEPES 10, and glucose 5 (pH was adjusted to 7.4 with NaOH). Cardiomyocytes were initially voltage-clamped (holding potential -80 mV) and dialyzed with an internal solution containing (in mM) KCl 120, CaCl_2_ 1.5, MgCl_2_ 5.5, Na_2_ATP 5, K_2_-EGTA 5, and HEPES 10 (pH was adjusted to 7.2 with KOH). APs were elicited at 0.5 Hz with rectangular pulses (5 ms at 125% threshold) in current-clamp mode. Elicited APs were allowed to stabilize before one or more sequences of ∼1 min each were acquired from each cell. AP recordings were digitized at a sampling frequency of 20 kHz. Electrophysiological data were analyzed off-line, where the resting membrane potential, amplitude, maximal upstroke velocity (dV/dt)_max_ and durations at 30, 50, and 90% repolarization were averaged from each sequence of APs.

I_Na_ in α1-syntrophin knockdown (*Snta1* fl/fl^+^) or α1-syntrophin wild-type littermate (*Snta1* fl/fl^-^) cardiomyocytes were carried out using an internal solution containing (in mM) CsCl 60, Cs-aspartate 70, CaCl_2_ 1, MgCl_2_ 1, HEPES 10, EGTA 11, and Na_2_ATP 5 (pH was adjusted to 7.2 with CsOH). Cardiomyocytes were bathed in a solution containing (in mM) NaCl 5, NMDG-Cl 125, CaCl_2_ 2, MgCl_2_ 1.2, CsCl 5, HEPES 10, and glucose 5 (pH was adjusted to 7.4 with CsOH). Nifedipine (10 μM) and cobalt chloride (CoCl_2_) (10 μM) were added to the extracellular solution in order to inhibit calcium currents.

To assess the functional consequences of detubulation, currents carried by the calcium channel Ca_v_1.2 (I_Ba_) were recorded using the internal pipette solution composed of (in mM) 60 CsCl, 70 Cs-aspartate, 1 MgCl_2_, 10 HEPES, 11 EGTA and 5 Mg-ATP (pH was adjusted to 7.2 with CsOH). The external solution contained (in mM) 5 NaCl, 125 NMDG-Cl, 5.6 CsCl, 5 BaCl_2_, 1 MgCl_2_, 10 HEPES and 5 D-glucose (pH was adjusted to 7.4 with CsOH).

I_to_ and I_K1_ recordings were performed using an internal solution containing (in mM): KCl 130, Mg-ATP 4, NaCl 12, MgCl_2_ 1, CaCl_2_ 1, EGTA 10, and HEPES 10 (pH was adjusted to 7.2 with KOH). Myocytes were bathed with a solution containing (in mM): NaCl 140, KCl 5.4, CaCl_2_ 1.8, MgCl_2_ 1, HEPES 10, and Glucose 5 (pH was adjusted to 7.4 with NaOH). Voltage-gated sodium channels were blocked with 50 μM of Tetrodotoxin (TTX). L-type voltage-gated calcium channels were blocked with 3 mM CoCl_2_. I_K1_ barium-sensitive current was calculated by subtracting potassium current recorded after perfusion of an extracellular solution containing 100 μM barium chloride (BaCl_2_) from potassium current recorded before application of BaCl_2_.To record I_to_, 100 μM BaCl_2_ was added to the extracellular solution to block I_K1_ current. I–V curves were not fitted.

Terminology: MaxI corresponds to the maximum current (in absolute value) recorded during the protocol used to determine the relationship between currents and membrane voltages (IV curve). For I_Na_, this is the maximum inward current recorded. For Ito, the maximum current is the outward current measured for a membrane voltage of +50 mV. For I_K1_, the maximum current is the inward current measured for a membrane voltage of -130 mV.

### Cell-Attached Electrophysiology

I_Na_ macro recordings were recorded in the cell-attached configuration either on the lateral membrane of cardiomyocytes or on plasma membrane of HEK-293 cells transiently expressing Na_v_1.4. All cell-attached recordings were performed at RT (22–23°C), using a low-noise Axopatch 200B amplifier (Molecular Devices, Sunnyvale, CA, United States). Borosilicate glass pipettes were pulled to a series resistance of ∼1.5 MΩ (diameter of opening ∼20 μm^2^). No statistical difference was noticed in averages pipette resistance between all conditions indicating that a similar membrane areas was patched. The internal pipette (intrapipette) solution was composed of (in mM) 148 NaCl, 1.8 CaCl_2_, 1 MgCl_2_, 5 D-glucose, and 10 HEPES (pH was adjusted to 7.4 with NaOH). The external solution, to depolarize the cell membrane ∼0 mV, contained (in mM) 125 KCl, 30 KOH, 10 EGTA, 2 CaCl_2_, and 5 HEPES (pH was adjusted to 7.15 with KOH). TTX-sensitive voltage-gated sodium channels (Na_v_1.1, Na_v_1.2, Na_v_1.3, Na_v_1.4, Na_v_1.6, and Na_v_1.7) were inhibited by 50 nM TTX in the intrapipette solution. A 4-pole Bessel low-pass filter at 2 kHz, was applied during the recording and a sampling rate of 20 kHz was used for the acquisition of the raw traces. P over 4 protocol was applied. pClamp software, version 8 (Axon Instruments, Union City, CA, United States) was used for recordings. Data were analyzed using pClamp software, version 8 (Axon Instruments) and OriginPro, version 7.5 (OriginLab Corporation, Northampton, MA, United States). I–V sodium currents curves were fitted with the equation, *y* = g^∗^(V_m_-V_rev_)/(1+exp[(V_m_-V_1/2_)/K]). To determine the voltage dependence of steady-state activation and inactivation curves, the data from individual cells were fitted with the Boltzmann equation, *y* = 1/(1+exp[(V_m_-V_1/2_)/K]). Y is the normalized current or conductance, V_m_ is the membrane potential, V_1/2_ is the voltage at which half of the channels are activated or inactivated, K is the slope factor, and V_rev_ the reversal potential.

Single channel barium recordings have been performed in cell-attached configuration. Average pipette size was 1.5 MΩ. Currents were measured at RT (22–23°C) using a low-noise Axopatch 200B amplifier (Molecular Devices, Sunnyvale, CA, United States). The internal pipette solution was composed of (in mM) 110 BaCl, 10 TEA-Cl, and 5 HEPES (pH was adjusted to 7.4 with NMDG-OH). To depolarize the cell membrane ∼0 mV, the external solution contained (in mM) 125 KCl, 30 KOH, 10 EGTA, 2 CaCl_2_, and 5 HEPES (pH was adjusted to 7.15 with KOH). A 4-pole Bessel low-pass filter at 2 kHz was applied during the recording at sampling rate of 20 kHz for the acquisition of raw traces. For each cell, a single depolarizing protocol at -10 mV from a holding potential at -120 mV was applied at least 150 times. Post-acquisition, data were filtered using a low pass Gaussian filter and analyzed using pClamp software, version 9.2 (Axon Instruments, Union City, CA, United States).

### Proximity Ligation Assay

Duolink^®^ (Olink Bioscience, Uppsala, Sweden) is a proximity ligation assay (PLA) that enables the visualization of protein-protein interactions allowing the *in situ* detection of epitopes that are near each other (<40 nm apart). Two primary antibodies raised in different species recognize the target antigen or antigens of interest. Species-specific secondary antibodies, called PLA probes, each with a unique short DNA strand attached to it, bind to the primary antibodies. When PLA probes are nearby, the DNA strands can interact through subsequent addition of two other circle-forming DNA oligonucleotides. After joining of the two oligonucleotides by enzymatic ligation, they are amplified via rolling circle amplification using a polymerase. After the amplification reaction, several-hundredfold replication of the DNA circle has occurred, and labeled complementary oligonucleotide probes highlight the product. The resulting high concentration of fluorescence in each single-molecule amplification product is easily visible as a distinct bright dot when viewed with a fluorescence microscope. Experiments were performed according to the manufacturer’s recommendations. Briefly, isolated mouse ventricular cardiac cells were fixed in 4% paraformaldehyde for 10 min and incubated twice for 7 min in PBS with 0.02% Triton X-100 at RT. Subsequently, cells were incubated 15 min in blocking solution containing 1% bovine serum albumin (BSA). Cardiomyocytes from α1-syntrophin knockdown or α1-syntrophin wild-type littermate were then incubated with commercially available mouse monoclonal anti-pan-syntrophin and polyclonal anti-Na_v_1.5 primary antibodies as specified below at a dilution of 1/100 and 1/200, respectively. Subsequent incubation steps were performed at 37°C and the appropriate washing buffers were used after each incubation, as specified in the protocol. After washing 3 times in PBS to remove the primary antibody, Duolink^®^ secondary antibodies conjugated to PLA probes were added to the tissue for a 1 h incubation. These PLA probes consist of oligonucleotides that were subsequently joined together in a circle after the addition of a ligation solution (containing ligase enzyme) and incubation for 30 min. Next, the rolling circle amplification of this circular template was achieved by the addition of an amplification solution, containing polymerase, and fluorescently labeled oligonucleotides, which hybridized to the circular template during a 100 min incubation. Samples were then mounted with Duolink^®^ *in situ* mounting medium with DAPI (1 μl/200 μl PBS) and viewed with a confocal microscope (LSM710, Zeiss, Germany).

### Protein Extraction and Western Blots

Whole hearts were extracted from mice and the atria were removed before homogenization of the ventricles in 1.5 mL lysis buffer (50 mM HEPES, 150 mM NaCl, 1 mM EGTA, 10% glycerol, 1.5 mM MgCl_2_, and Complete^®^ protease inhibitor cocktail from Roche, Basel, Switzerland) using a Polytron. Triton X-100 was then added to make a final concentration of 1% (final volume 3 mL) before lysis of the samples on a rotating wheel for 1 h at 4°C. Soluble fractions of the mouse heart were obtained by subsequent centrifugation for 15 min at 13,000 rpm at 4°C. To determine the correct volume of sample needed to load equivalent amounts of protein on a gel, the protein concentration of each of the lysate samples was measured in triplicate by Bradford assay and interpolated by a BSA standard curve. Samples were denatured at 95°C for 5 min before loading them on a gel. To analyze and compare protein content in whole mouse hearts, 25 or 50 μg of ventricular mouse heart lysate samples were loaded on a 1.0 mm-thick 10% Tris–acetate gel for high molecular weight proteins (Invitrogen). Gels were run at 150 V for 1 h and then subsequently transferred to nitrocellulose membranes using the Biorad Turbo Transfer System (Biorad, Hercules, CA, United States). All membranes were stained with Ponceau as a qualitative check for equivalent loading of total protein. Membranes were then rinsed twice with PBS before using the SNAP i.d. system (Millipore, Zug, Switzerland) for western blotting. 1% BSA was used as a blocking solution and incubated with membranes for 10 min before incubation with the primary antibodies for 10 min. Membranes were subsequently washed four times in PBS + 0.1% Tween before incubating with fluorescent secondary antibodies for 10 min. After four more washes with PBS + 0.1% Tween and three washes in PBS, membranes were scanned with the Odyssey^®^ Infrared Imaging System (LI-COR Biosciences, Bad Homberg, Germany) for detection of fluorescent protein.

### Immunocytochemistry

Following cardiomyocyte isolation, cells from α1-syntrophin knockdown and α1-syntrophin wild-type littermate hearts were fixed with cold methanol at -20°C for 10 min. Cells were subsequently washed twice in PBS before permeabilization with PBS containing 0.02% Triton X-100 for 14 min. Then, the cardiomyocytes were blocked for 15 min at RT with 10% goat serum in TBS containing 2.3% of fab fragment donkey anti-mouse IgG (H+L) (Jackson Immuno Research, Baltimore, United States) at 1.3 mg/mL. Cardiomyocytes were then incubated overnight with primary antibodies diluted in 3% goat serum in TBS containing 0.02% Triton X-100. Afterward, cells were washed four times with 3% goat serum in TBS for 5 min each, and then incubated with secondary antibodies for 2 h at RT. Cells were then washed four times in PBS (5 min each). Finally, DAPI solution (1 μl/200 μl PBS) was applied on cardiac cells for 20 min at RT. Before examination with the confocal microscope (LSM710, Zeiss, Germany), cardiomyocytes were embedded in FluorSave^TM^ Reagent (Calbiochem). Total signal intensity was calculated using Zen 2.1 software version 11 from Carl Zeiss (Germany).

### Antibodies

For western blots, the antibody against pan-syntrophin (MAI-745, Affinity BioReagents, Golden, CO, United States) was used at a dilution of 1/200. A custom rabbit polyclonal antibody against α1-syntrophin (Pineda Antibody Service, Berlin) was used at a dilution of 1/1000. The antibodies against Cre (69050; Novagen, EMD Millipore-MERK, Schaffhausen, Switzerland) was used at a dilution of 1/500 and the antibody against calnexin (C4731; Sigma-Aldrich, St. Louis, MO, United States) was used at 1/1000. For Duolink^®^ experiments, the antibody against syntrophin (pan-syntrophin, MAI-745, Affinity BioReagents, Golden, CO, United States) was used at a dilution of 1/100 and custom rabbit polyclonal antibody against Na_v_1.5 (Pineda Antibody Service, Berlin) was used at a dilution of 1/200. For staining, the anti-pan-syntrophin antibody (MAI-745, Affinity BioReagents, Golden, CO, United States) was used at a dilution of 1/200. The antibody against Cre (69050; Novagen, EMD Millipore-MERK, Schaffhausen, Switzerland) was used at a dilution of 1/1000.

### Drugs

TTX was purchased from Alomone Labs (Jerusalem, Israel) and dissolved in intrapipette solution. The μ-conotoxin CnIIIC was purchased from Smartox (Saint Martin d’Heres, France) and dissolved in water.

### Statistical Analyses

Data are represented as means ± S.E.M. Statistical analyses were performed using Prism7 GraphPad^TM^ software. A Mann–Whitney *U* two-tailed test was used to compare two groups due to the small samples size. *p* < 0.05 was considered significant.

## Results

### Characterization of a Cardiac-Specific α1-Syntrophin Knockdown Mouse Tissue and Cells

**FIGURE 1:**
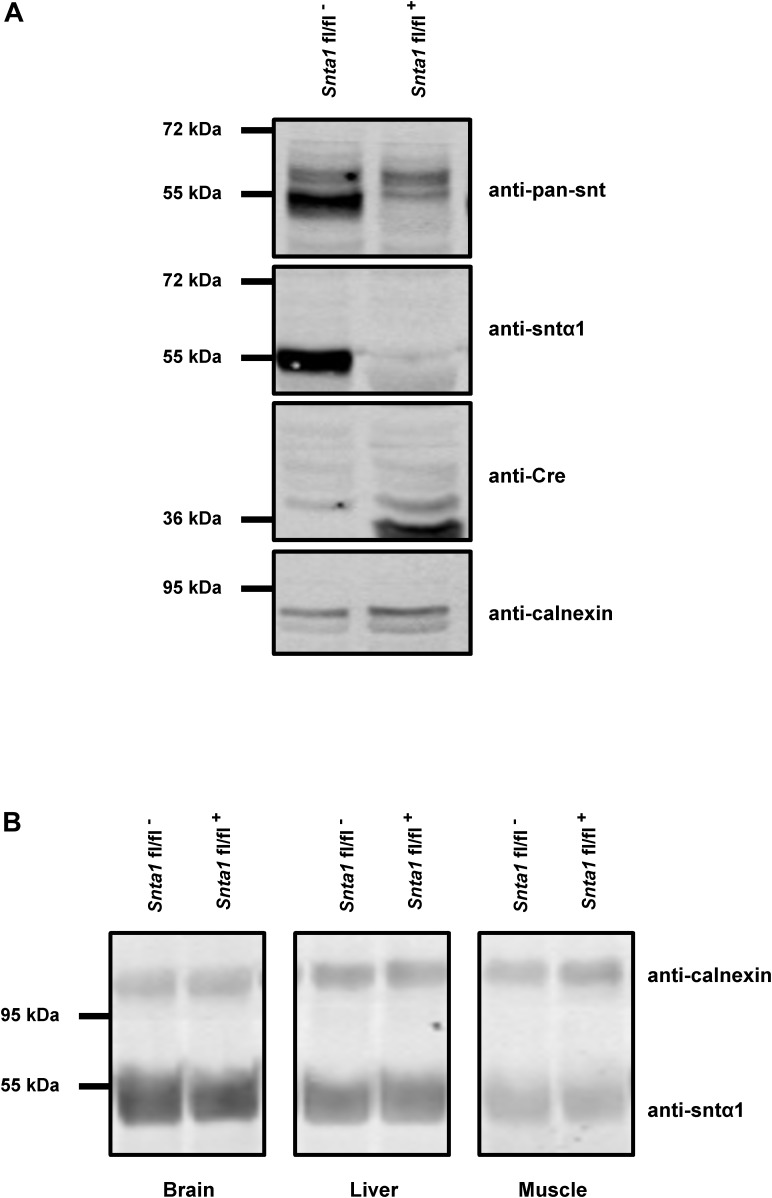
α1-Syntrophin expression in wild-type and knockdown α1-syntrophin knockdown tissues. **(A)** Western blots of whole hearts from wild-type (*Snta1* fl/fl^-^) and α1-syntrophin cardiac specific knockdown (*Snta1* fl/fl^+^) mice show a significant decrease of the α1-syntrophin protein (anti-Sntα1 antibody) and the absence of compensatory effects *via* other syntrophin isoforms (anti-pan-syntrophin (Snt) antibody). **(B)** Western blots from brain, liver and muscle from wild-type (*Snta1* fl/fl^-^) and α1-syntrophin cardiac specific knockdown (*Snta1* fl/fl^+^) mice show no effect on α1-syntrophin protein in other organs than the heart.

**FIGURE 2:**
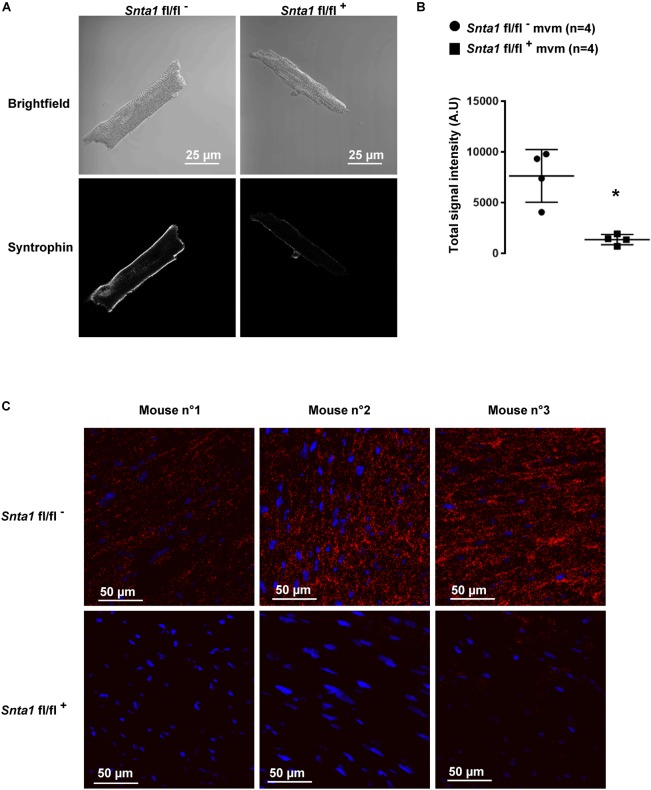
α1-Syntrophin staining in wild-type and knockdown α1-syntrophin knockdown cardiac sections and cardiomyocytes. **(A)** Immunostaining, using a pan-syntrophin antibody, of syntrophin in wild-type (*Snta1* fl/fl^-^) and cardiac-specific α1-syntrophin knockdown murine cardiomyocytes (mvm) (*Snta1* fl/fl^+^). **(B)** Intensity quantification of syntrophin signal of four cells confirming the significant decrease of syntrophin in knockdown mice. (^∗^*p* < 0.05). The number of cells is indicated in parentheses. **(C)** Duolink^®^ experiments showing a significant decrease in interaction (red dots) between Na_v_1.5 and syntrophin in α1-syntrophin cardiac specific knockdown cardiomyocytes (*Snta1* fl/fl^+^) compared to wild-type (*Snta1* fl/fl^-^).

**FIGURE 3:**
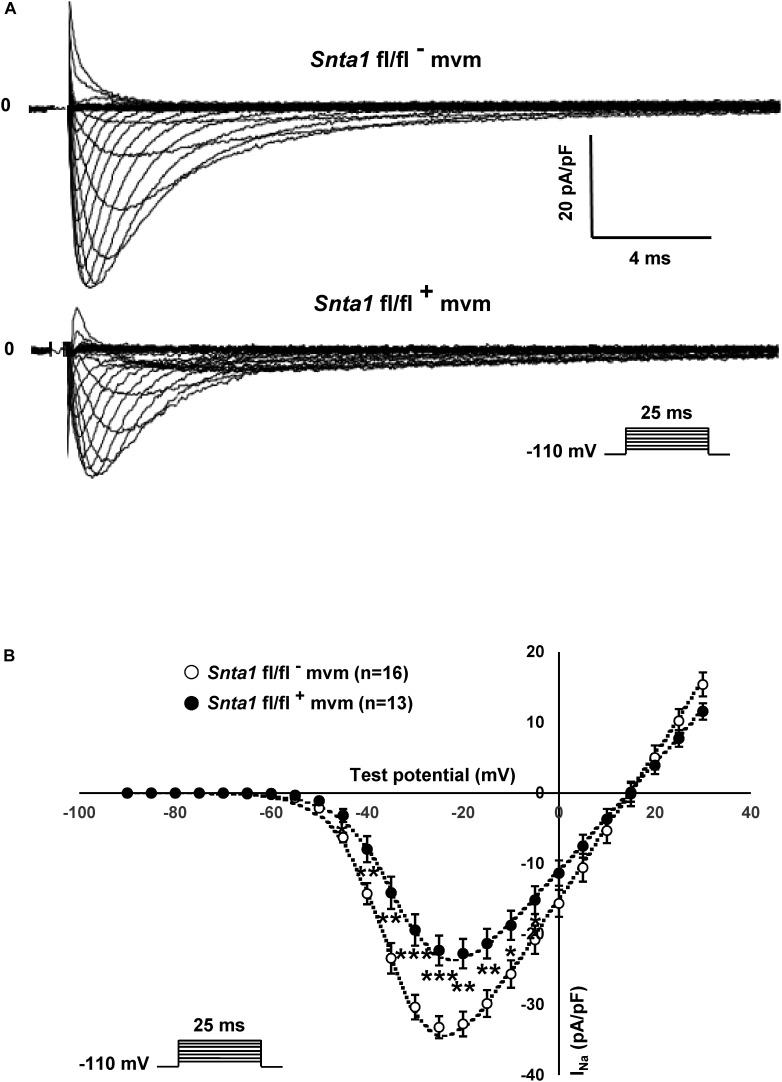
Sodium current measurements in in wild-type and knockdown α1-syntrophin knockdown cardiomyocytes. **(A)** Raw traces of whole-cell sodium currents recorded in adult ventricular cardiomyocytes from wild-type (*Snta1* fl/fl^-^) and α1-syntrophin cardiac specific knockdown cardiomyocytes (*Snta1* fl/fl^+^). **(B)**, Whole-cell sodium current I–V curves show a significant decrease in the current density in knockdown cardiomyocytes compared to wild-type littermate cells. (^∗^*p* < 0.05, ^∗∗^*p* < 0.01, ^∗∗∗^*p* < 0.001). The number of cells is indicated in parentheses.

**FIGURE 4:**
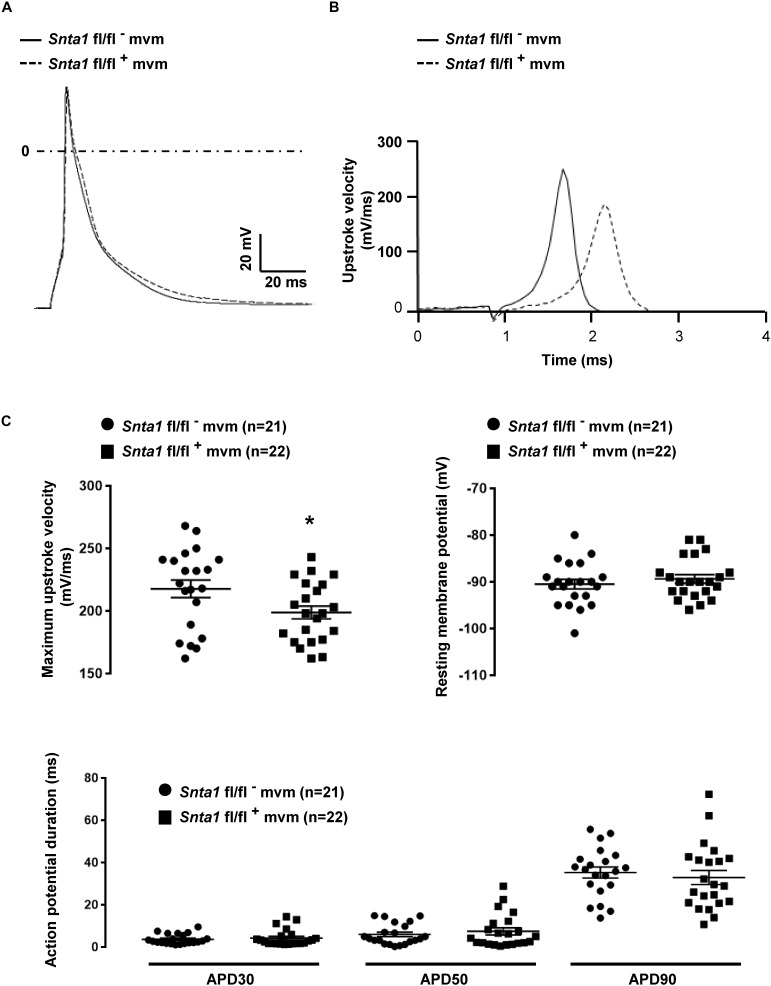
Cardiac action potentials measurements in in wild-type and knockdown α1-syntrophin knockdown cardiomyocytes. **(A)** Raw traces of cardiac action potential recorded in wild-type adult ventricular cardiomyocytes (*Snta1* fl/fl^-^) and α1-syntrophin cardiac specific knockdown cardiomyocytes (*Sntα1* fl/fl^+^). **(B)** Upstroke velocity of cardiac action potential recorded in **A** from wild-type adult ventricular cardiomyocytes (*Snta1* fl/fl^-^) and α1-syntrophin cardiac specific knockdown cardiomyocytes (*Sntα1* fl/fl^+^) showing the slowing of the upstroke velocity in action potential from knockdown cardiomyocytes. **(C)** Cardiac action potential parameters measured in wild-type (*Snta1* fl/fl^-^) and α1-syntrophin cardiac specific knockdown cardiomyocytes (*Snta1* fl/fl^+^) reveal the significant decrease of the maximal upstroke velocity.

**FIGURE 5:**
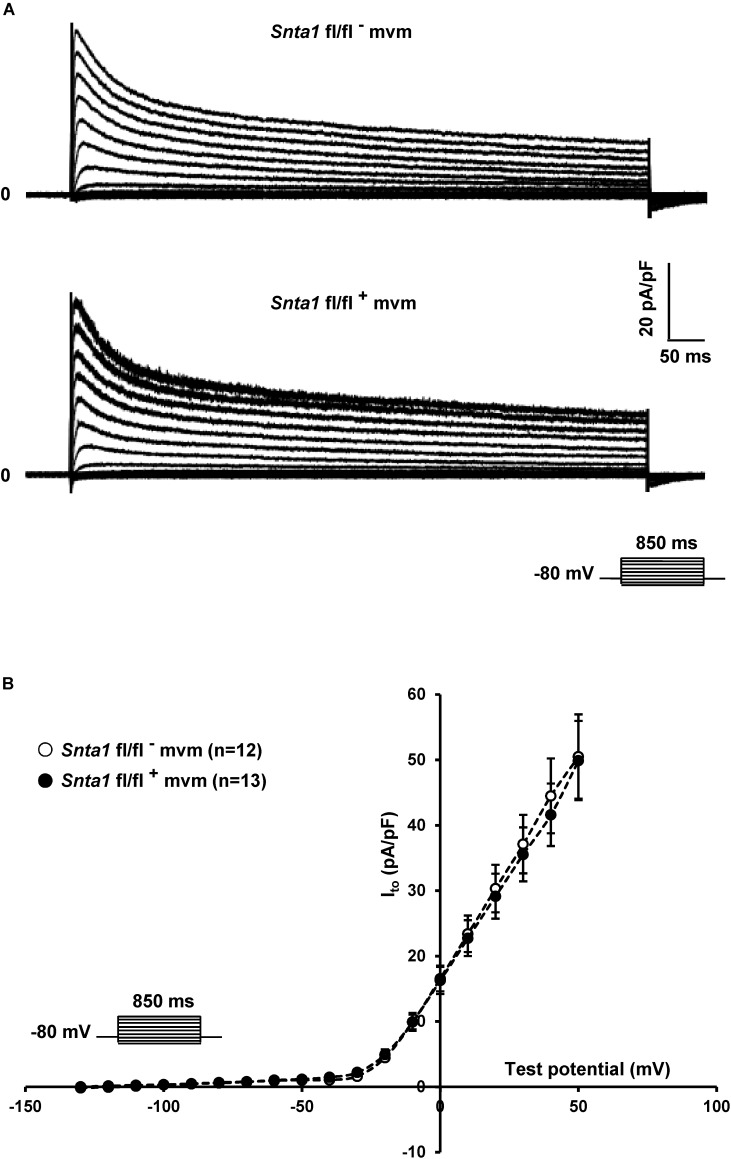
Potassium current I_to_ measurements in wild-type and knockdown α1-syntrophin knockdown cardiomyocytes. **(A,B)** Raw traces and potassium current I–V relationships show that knockdown of α1-syntrophin has no effect on these parameters in cardiac cells. The number of cells is indicated in parentheses. The dashed lines are drawn to connect data points and do not represent lines of best fit.

**FIGURE 6:**
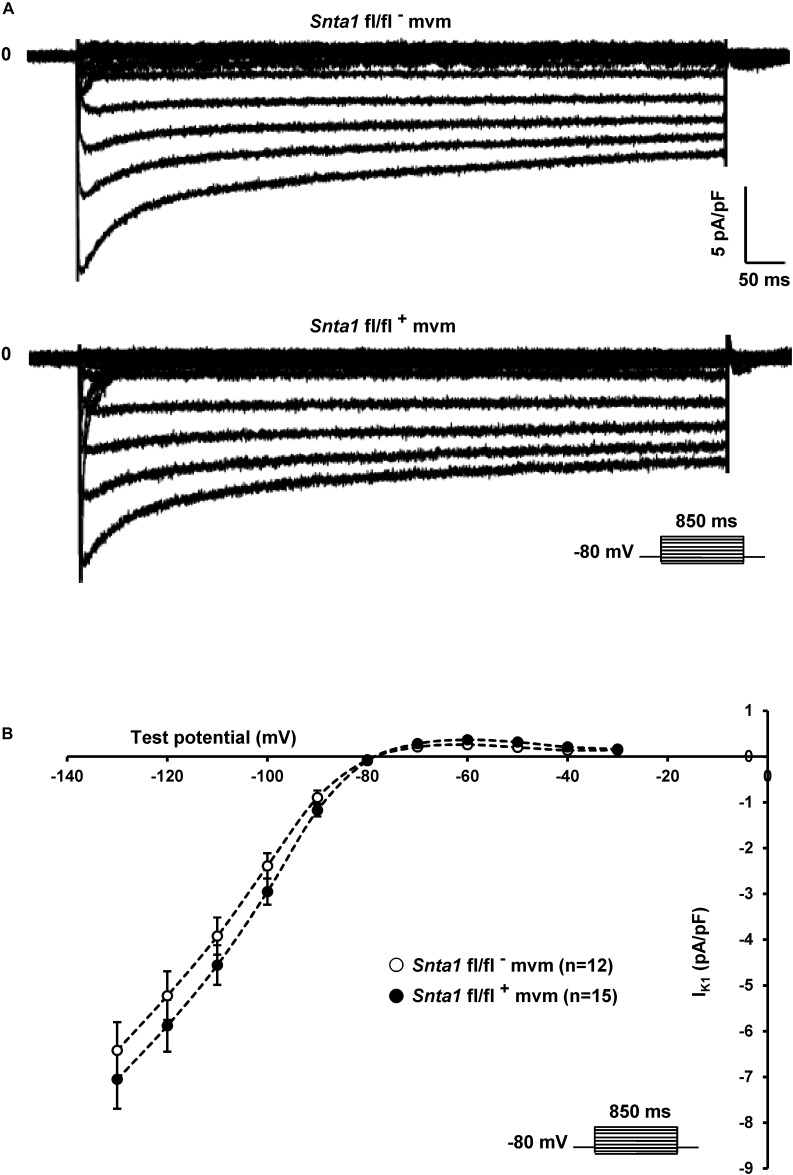
Potassium current I_K1_ measurements in wild-type and knockdown α1-syntrophin knockdown cardiomyocytes. **(A,B)** Raw traces and potassium current I–V relationships show that knockdown of α1-syntrophin had no effect on these parameters in cardiac cells. The number of cells is indicated in parentheses. The dashed lines are drawn to connect data points and do not represent lines of best fit.

The remaining I_Na_ at the lateral membrane of Na_v_1.5 ΔSIV mouse cardiomyocytes (Shy et al., 2014) raised the question which molecular determinants are involved in this lateral membrane pool of Na_v_1.5 channels. Although the Na_v_1.5 SIV motif mediates the interaction with α1-syntrophin of the dystrophin–syntrophin complex, Na_v_1.5 contains a similar motif (Ser-Leu-Ala) in its N-terminal domain, which was previously shown to interact with α1-syntrophin (Matamoros et al., 2016). Motivated by these observations, we generated a cardiac-specific α1-syntrophin knockdown mouse strain (described in Supplementary Figure S1) and investigated the consequences of α1-syntrophin knockdown on the I_Na_ of ventricular cardiomyocytes. Using either pan-syntrophin (targetting all members of the syntrophin family) or α1-syntrophin antibodies, western blots confirmed the cardiac-specific reduction of the α1-syntrophin expression in *Snta1* fl/fl^+^ cardiac tissues compared to the control *Snta1* fl/fl^-^ (Figure 1A,B). In addition, immunostaining experiments using the pan-syntrophin antibody showed a significant decrease of syntrophin expression in isolated ventricular cardiomyocytes *Snta1* fl/fl^+^ compared to *Snta1* fl/fl^-^ cardiac cells (Figure 2A,B). Finally, PLA experiments showed a substantial decrease of the interaction (based on the absence of red dots) between Na_v_1.5 and α1-syntrophin in *Snta1* fl/fl^+^ cardiomyocytes compared to the control, suggesting that α1-syntrophin and Na_v_1.5 no longer interact in α1-syntrophin knockdown cells (Figure 2C). In parallel to the molecular characterization, functional investigations were performed. Whole-cell I_Na_ was recorded in freshly isolated ventricular cardiomyocytes. As shown in Figure 3A,B, comparing knockdown cardiomyocytes to the wild-type cells, a significant reduction of the I_Na_ was observed (*Snta1* fl/fl^-^ Max inward I_Na_: 33 ± 2 pA/pF, *n* = 16; *Snta1* fl/fl^+^ Max inward I_Na_: 22 ± 2 pA/pF, *n* = 13, *p* < 0.05), without alteration of either cell membrane capacitance (*Snta1* fl/fl^-^: 166 ± 8 pF, *n* = 16; *Snta1* fl/fl^+^: 159 ± 12 pF, *n* = 13, *p* > 0.05) or I_Na_ biophysical properties (Table 2, Figure 3A,B, and Supplementary Figure S2). Cardiac AP recordings moreover did not show any alteration of neither the resting membrane potential nor AP duration (Table 1 and Figure 4A,C). Only the upstroke velocity was significantly decreased in α1-syntrophin knockdown cells compared to controls (Table 1 and Figure 4B,C). Using heterologous expression systems, (Matamoros et al., 2016) have also demonstrated that α1-syntrophin interacts with K_v_4.2/4.3 and Kir2.1 potassium channels, which conduct the I_to_ and I_K1_ currents, respectively. Although the decrease of the upstroke velocity of the AP suggests that only I_Na_ is altered in α1-syntrophin knockdown conditions, we recorded the I_to_ and I_K1_ currents in the absence of α1-syntrophin. As shown in Figure 5, 6, no alteration was observed in α1-syntrophin knockdown cells compared to the control for both currents (*Snta1* fl/fl^-^ MaxI_to_ at +50 mV: 51 ± 6 pA/pF [176 ± 11 pF], *n* = 12; *Snta1* fl/fl^+^ MaxI_to_ at +50 mV: 50 ± 6 pA/pF [151 ± 11 pF], *n* = 13, *p* > 0.05; Figure 5A,B), and (*Snta1* fl/fl^-^ MaxI_K1_ at -130 mV: 6.4 ± 0.6 pA/pF [183 ± 16 pF], *n* = 12; *Snta1* fl/fl^+^ MaxI_K1_ at -130 mV: 7.0 ± 0.6 pA/pF [159 ± 10 pF], *n* = 15, *p* > 0.05; Figure 6A,B) suggesting that mainly the Na_v_1.5-mediated current is down-regulated in this α1-syntrophin knockdown model.

**Table 1:** Cardiac action potential parameters.

	*Sntα1* fl/fl^-^	*Sntα1* fl/fl^+^
RMP (mV)	-90 ± 1 (*n* = 21)	-89 ± 1 (*n* = 22)
APD_30_ (ms)	3.6 ± 0.5 (*n* = 21)	4.2 ± 0.8 (*n* = 22)
APD_50_ (ms)	7.6 ± 1.1 (*n* = 21)	9.1 ± 1.7 (*n* = 22)
APD_90_ (ms)	35.5 ± 2.6 (*n* = 21)	33.2 ± 3.3 (*n* = 22)
dV/dt (mV/ms)	218 ± 7 (*n* = 21)	199 ± 5 (*n* = 22)^∗^

*RMP, resting membrane potential; APD, action potential duration and maximum upstroke velocity (dV/dt) calculated from ventricular cardiac action potentials recorded in wild-type (Snta1 fl/fl^-^) and syntrophin knockdown (Snta1 fl/fl^+^) cardiomyocytes. Number of cells in parenthesis. ^∗^p < 0.05.*

### I_Na_ at the Lateral Membrane of α1-Syntrophin Knockdown Cardiomyocytes

**FIGURE 7:**
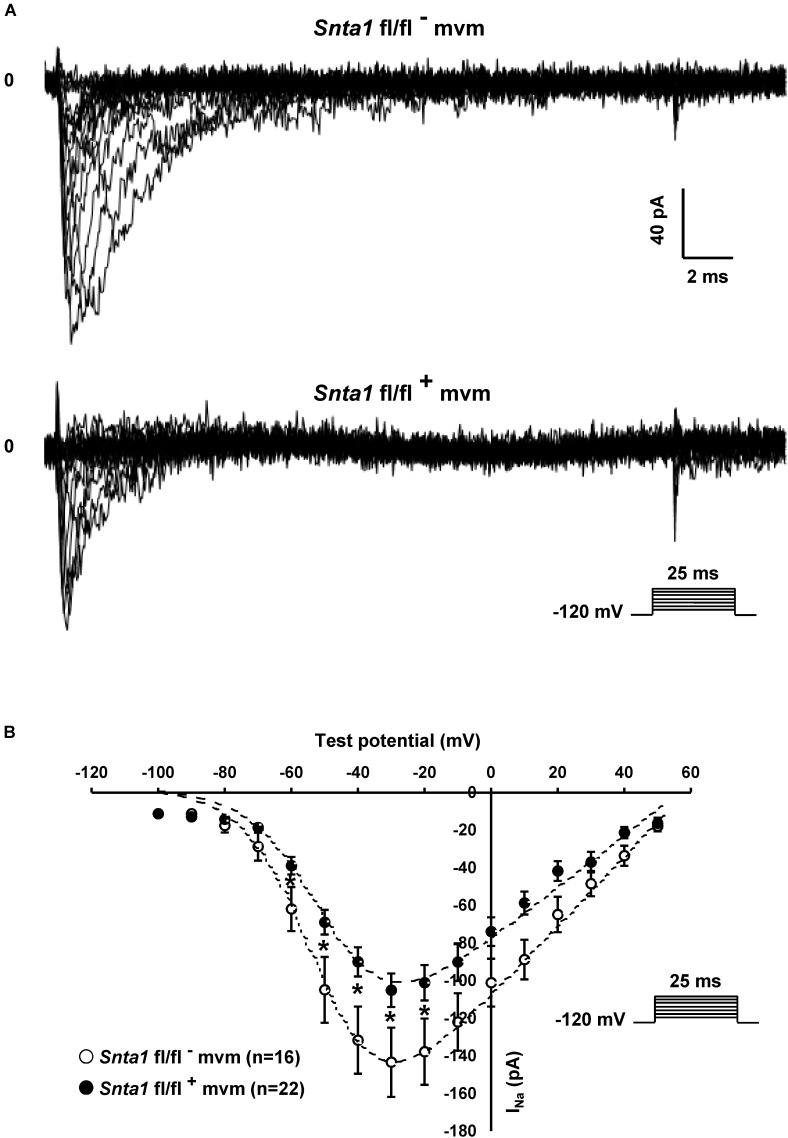
Lateral sodium currents measurements in cell-attached configuration in wild-type and α1-syntrophin knockdown cardiomyocytes. **(A)**, Raw traces of cell-attached sodium currents recorded from adult ventricular cardiomyocytes from wild-type (*Snta1* fl/fl^-^) and α1-syntrophin cardiac specific knockdown cardiomyocytes (*Snta1* fl/fl^+^). **(B)** Sodium current I–V curves from α1-syntrophin cardiac specific knockdown cardiomyocytes (*Snta1* fl/fl^+^) (∙) reveal a decrease of the lateral sodium current compared to the control (*Snta1* fl/fl^-^) (∘).

Using the cell-attached patch-clamp configuration, we then recorded the I_Na_ specifically at the lateral membrane of α1-syntrophin knockdown cardiomyocytes. As shown in Figure 7A,B, using an intrapipette solution with 50 nM of TTX to block TTX-sensitive sodium channels (see section “Materials and Methods”), a sizable TTX-resistant sodium current was recorded in α1-syntrophin knockdown myocytes (Figure 7A,B). This I_Na_ is, however, smaller (without any alteration of the main biophysical properties) than the currents recorded in control cells (*Snta1* fl/fl^-^ Max inward I_Na_: 143 ± 18 pA, *n* = 16; *Snta1* fl/fl^+^ Max inward I_Na_: 105 ± 9 pA, *n* = 22, *p* < 0.05; glass pipette resistances: *Snta1* fl/fl^-^: 1.41 ± 0.08 MΩ, *n* = 16; *Snta1* fl/fl^+^: 1.45 ± 0.05 MΩ, *n* = 22, *p* > 0.05; Table 2, Figure 7A,B, and Supplementary Figure S3). Altogether, these results suggest that the reduction of α1-syntrophin expression in ventricular cardiomyocytes leads to a decrease of lateral membrane Na_v_1.5 currents similar to the findings reported by Shy et al. (2014) in ΔSIV mice.

**Table 2:** Activation and steady state inactivation curve parameters.

	Whole-cell		Cell-attached		Cell-attached	
	*Snta1* fl/fl^-^	*Snta1* fl/fl^+^	*Snta1* fl/fl^-^	*Snta1* fl/fl^+^	wild-type	ΔSIV
**Activation**						
K	5.5 ± 0.3 (*n* = 16)	5.3 ± 0.3 (*n* = 13)	10.3 ± 0.5 (*n* = 16)	12.6 ± 2.6 (*n* = 22)	11.4 ± 1.2 (*n* = 8)	11.3 ± 0.9 (*n* = 11)
V_1/2_ (mV)	-33.4 ± 0.9 (*n* = 16)	-31.3 ± 1.2 (*n* = 13)	-46.7 ± 1.9 (*n* = 16)	-48.5 ± 1.1 (*n* = 22)	-46.4 ± 2.4 (*n* = 8)	-51.3 ± 1.4 (*n* = 11)
**Inactivation**						
K	5.5 ± 0.2 (*n* = 15)	6.2 ± 0.1 (*n* = 12)^∗^	8.4 ± 0.4 (*n* = 24)	9.0 ± 0.7 (*n* = 20)	8.4 ± 1.2 (*n* = 6)	9.4 ± 1.0 (*n* = 9)
V_1/2_ (mV)	-78.4 ± 1.0 (*n* = 15)	-76.6 ± 1.1 (*n* = 12)	-93.0 ± 1.0 (*n* = 24)	-94.5 ± 0.9 (*n* = 20)	-102.2 ± 2.1 (*n* = 6)	-100.5 ± 1.3 (*n* = 9)

*Slope (K) and V_1/2_ calculated from ventricular sodium current recorded in wild-type (Snta1 fl/fl^-^), syntrophin knockdown (Snta1 fl/fl^+^), and ΔSIV cardiomyocytes. Number of cells in parenthesis. ^∗^p < 0.05.*

### I_Na_ From α1-Syntrophin Knockdown and ΔSIV Cardiomyocytes

**FIGURE 8:**
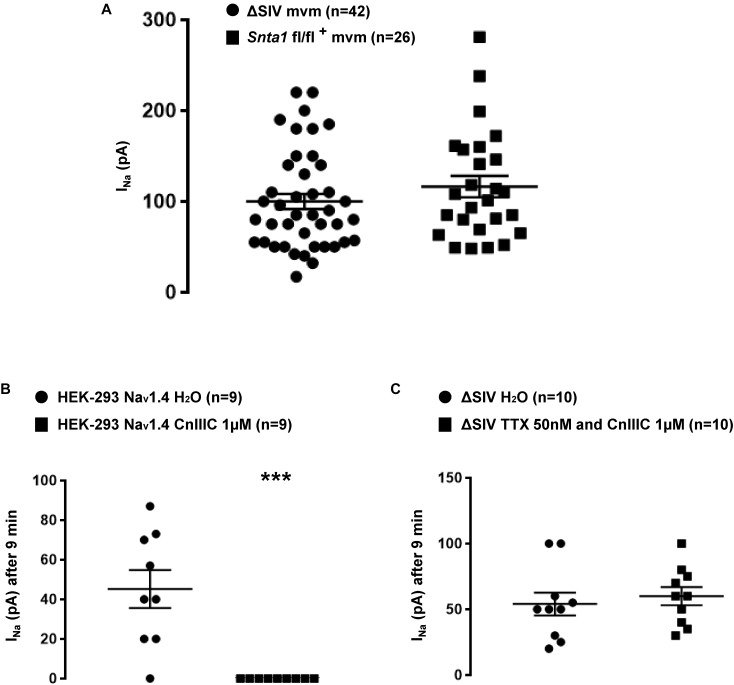
Pharmacological characterization of the remaining sodium current at the lateral membrane of cardiomyocytes. **(A)** Plot chart showing the similarity in average sodium currents recorded at the lateral membrane of cardiomyocytes from ΔSIV and α1-syntrophin cardiac specific knockdown (*Snta1* fl/fl^+^) cardiomyocytes using cell-attached configuration. **(B)** μ-conotoxin CnIIIC (CnIIIC) mediated the reduction in Na_v_1.4 I_Na_ after 9 min of application in HEK-293 cell line. **(C)** Tetrodotoxin (TTX) and μ-conotoxin CnIIIC (CnIIIC) did not mediate any effect on I_Na_ recorded on the lateral membrane of ΔSIV cardiomyocytes using the cell-attached configuration (^∗^*p* < 0.05, ^∗∗^*p* < 0.01, ^∗∗∗^*p* < 0.001). The number of cells is indicated in parentheses.

We then performed I_Na_ recordings using the cell-attached patch-clamp configuration on isolated ventricular cardiomyocytes from wild-type and ΔSIV mice. Again, similar to the observations by Shy et al. (2014), the TTX-resistant maximum I_Na_ at the lateral membrane was significantly decreased in ΔSIV cardiomyocytes compared to controls (wild-type Max inward I_Na_: 198 ± 39 pA, *n* = 10; ΔSIV Max inward I_Na_: 107 ± 14 pA, *n* = 13, *p* < 0.05; glass pipette resistances: wild-type: 1.33 ± 0.09 MΩ, *n* = 10; ΔSIV: 1.46 ± 0.04 MΩ, *n* = 13, *p* > 0.05; Supplementary Figure S4). As already reported, the biophysical properties (steady-state inactivation and activation curves) did not differ between wild-type and ΔSIV I_Na_ (Table 2 and Supplementary Figure S5; Shy et al., 2014). Of note, the peak I_Na_ also did not differ significantly between lateral membranes of ΔSIV and α1-syntrophin knockdown cardiomyocytes (ΔSIV Max inward I_Na_: 100 ± 8 pA, *n* = 42; *Snta1* fl/fl^+^ Max inward I_Na_: 116 ± 12 pA, *n* = 26, *p* > 0.05) (Figure 8A). In summary, these results suggest that a sizable fraction of the TTX-resistant I_Na_ at the lateral membrane of ventricular cardiomyocytes depends neither on the Na_v_1.5 SIV motif nor on α1-syntrophin.

### Na_v_1.5 Channels and the Remaining I_Na_ at the Lateral Membrane

Several studies reported that other Na_v_1.*x* isoforms may be expressed in cardiac cells in addition to Na_v_1.5, such as Na_v_1.1 (Westenbroek et al., 2013), Na_v_1.2 (Stoetzer et al., 2016), Na_v_1.3 (Westenbroek et al., 2013), Na_v_1.4 (Westenbroek et al., 2013), Na_v_1.6 (Westenbroek et al., 2013), and Na_v_1.8 (Yang et al., 2012). Although we performed the aforementioned I_Na_ recordings in the presence of 50 nM TTX, which efficiently inhibits Na_v_1.1 (IC_50_ ∼10 nM), Na_v_1.2 (IC_50_ ∼10 nM), Na_v_1.3 (IC_50_ ∼10 nM), and Na_v_1.6 (IC_50_ ∼5 nM), I_Na_ from Na_v_1.4 (IC_50_ ∼25 nM) and Na_v_1.8 (IC_50_ > 50 μM) could still be present. As shown in Supplementary Figure S4, in recordings from ΔSIV cells, the I–V relationship presents the maximum I_Na_ at a membrane voltage of around -30 mV. This suggests that the majority of this remaining current is not carried by Na_v_1.8, whose maximum current lies at more depolarized values (around 0 mV) (Stroud et al., 2016). We then addressed the question whether Na_v_1.4 contributes to the remaining current. HEK-293 cells were transfected with a Na_v_1.4 construct, and currents were recorded using the cell-attached patch-clamp approach in the presence of several drugs. As shown in Supplementary Figure S6A, I_Na_ was only recorded in cells transfected with Na_v_1.4 (Supplementary Figure S6A). Addition of 50 nM TTX in the intrapipette solution strongly decreased the Na_v_1.4 I_Na_ after 3 min of pipette seal (H_2_O Max inward I_Na_: 54 ± 21 pA, *n* = 7; TTX 50 nM Max inward I_Na_: 6 ± 3 pA, *n* = 9, *p* < 0.05) (Supplementary Figure S6B). In parallel, 9 min after pipette seal, an intrapipette solution containing 1 μM μ-conotoxin CnIIIC, a specific blocker of Na_v_1.4 (IC_50_ ∼1.4 nM) compared to Na_v_1.5 (IC_50_ > 10 μM) (Favreau et al., 2012), completely abolished Na_v_1.4 currents (H_2_O Max inward I_Na_: 45 ± 10 pA, *n* = 9; CnIIIC 1 μM Max inward I_Na_: 0 ± 0 pA, *n* = 9, *p* < 0.05; Figure 8B). Based on these observations, an intrapipette cocktail solution containing 50 nM of TTX and 1 μM μ-conotoxin CnIIIC was then used to repeat the I_Na_ recordings from the lateral membrane of ΔSIV cardiomyocytes. As shown in Figure 8C, 9 min after sealing the pipette, no alteration of the I_Na_ amplitude was observed (H_2_O Max inward I_Na_: 54 ± 9 pA, *n* = 10; cocktail Max inward I_Na_: 60 ± 10 pA, *n* = 10, *p* > 0.05; Figure 8C). These results suggest that the remaining lateral membrane I_Na_ in ΔSIV cardiomyocytes is mainly carried by Na_v_1.5 channels.

### Role of T-Tubules in Generating the Lateral Membrane I_Na_

**FIGURE 9:**
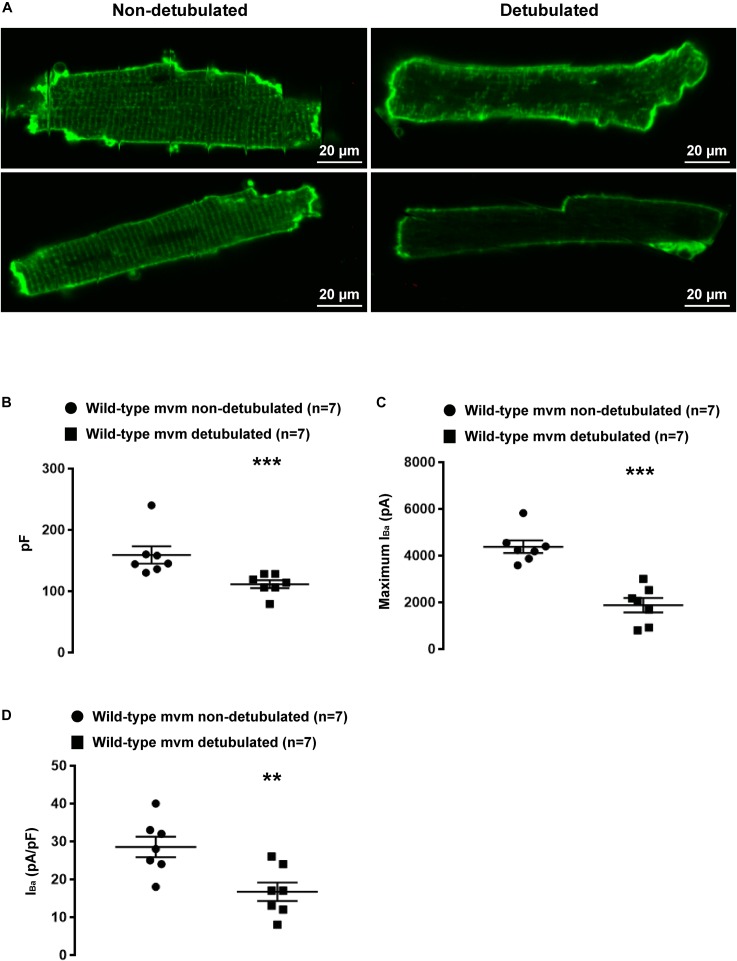
Validation of the detubulation procedure. **(A)** Confocal images showing detubulation mediated by formamide treatment on living cardiomyocytes. **(B)** Effect of detubulation on cell capacitance (pF). **(C,D)** functional consequences of detubulation on barium currents (I_Ba_) carried by calcium channels. (^∗∗^*p* < 0.01, ^∗∗∗^*p* < 0.001). The number of cells is indicated in parentheses.

**FIGURE 10:**
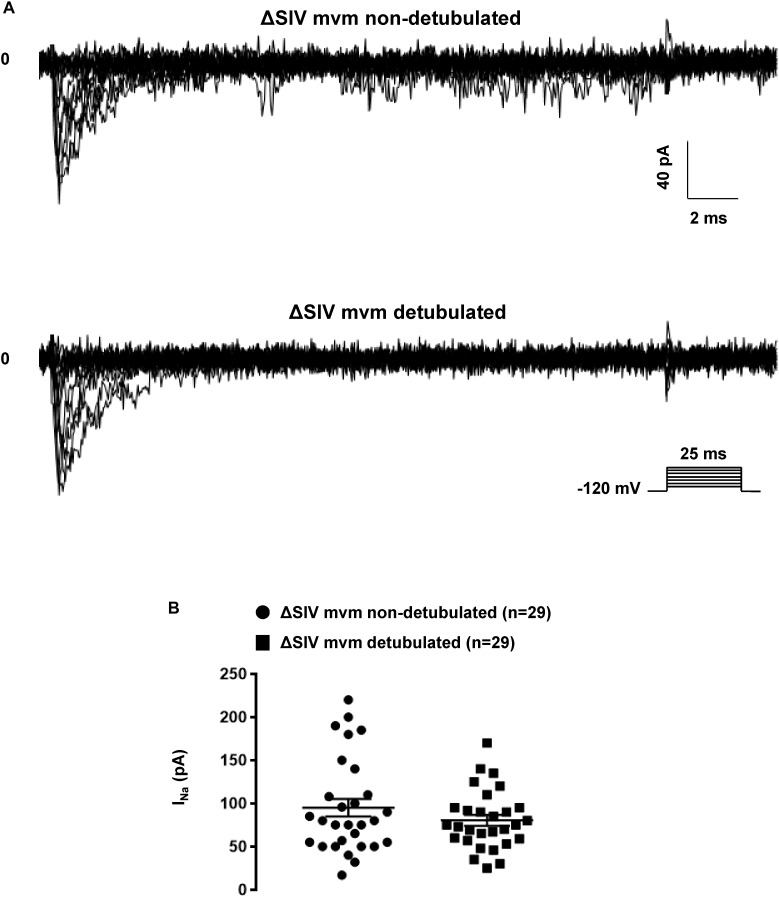
Effects of the detubulation on lateral sodium current. **(A)** Raw traces of cell-attached sodium currents recorded from non-detubulated and detubulated ΔSIV adult ventricular cardiomyocytes. **(B)** Consequences on peak current amplitudes of detubulation on lateral sodium currents from ΔSIV cardiomyocytes. The number of cells is indicated in parentheses.

We then aimed to investigate whether lateral membrane I_Na_ is of T-tubular origin, following a protocol described by Brette et al. (2002) to detubulate cardiac ventricular cells. As shown in Figure 9A, di-8-ANEPPS staining on living non-detubulated wild-type mouse cardiomyocytes presented a striated pattern reflecting the presence of T-tubules (Figure 9A). This striated pattern was absent in detubulated wild-type cells, validating the detubulation procedure (Figure 9A). To assess the functional consequences of detubulation, we first recorded the electrical capacitance of the cells, which approximates the surface of the plasma membrane, and, as a positive control, Ca_v_1.2-mediated currents using Ba^2+^ as charge carrier (Figure 9B–D). Detubulation significantly decreased the capacitance of the cardiac cells by 30 ± 4%, (non-detubulated: 159 ± 14 pF, *n* = 7; detubulated: 111 ± 6 pF, *n* = 7, *p* < 0.05; Figure 9B), and the maximum Ba^2+^ current by 57 ± 7% (non-detubulated Max inward I_Ba_: 4377 ± 270 pA, *n* = 7; detubulated Max inward I_Ba_: 1879 ± 306 pA, *n* = 7, *p* < 0.05; Figure 9C). Consequently, the Ba^2+^ current density (pA/pF) was also significantly decreased by 42 ± 9 % (non-detubulated Max inward I_Ba_: 29 ± 3 pA/pF, *n* = 7; detubulated Max inward I_Ba_: 17 ± 3 pA/pF, *n* = 7, *p* < 0.05; Figure 9D). These results validate the procedure of cardiomyocyte detubulation by formamide treatment. Then, lateral I_Na_ was recorded in non-detubulated and detubulated ΔSIV cardiomyocytes. As shown in Figure 10A,B, the maximum I_Na_ of detubulated cardiac cells compared to non-detubulated cardiomyocytes was not modified (non-detubulated Max inward I_Na_: 95 ± 10 pA, *n* = 29; detubulated Max inward I_Na_: 80 ± 6 pA, *n* = 29, *p* > 0.05; Figure 10A,B). These findings suggest that the remaining lateral membrane I_Na_ in ΔSIV myocytes is not carried by Na_v_1.5 channels in the T-tubular system, but rather by a distinct lateral membrane pool of Na_v_1.5 channels that is independent of the PDZ-binding motif SIV.

### Assessing the T-Tubular Pool of Ion Channels Using the Cell-Attached Patch Clamp Configuration

**FIGURE 11:**
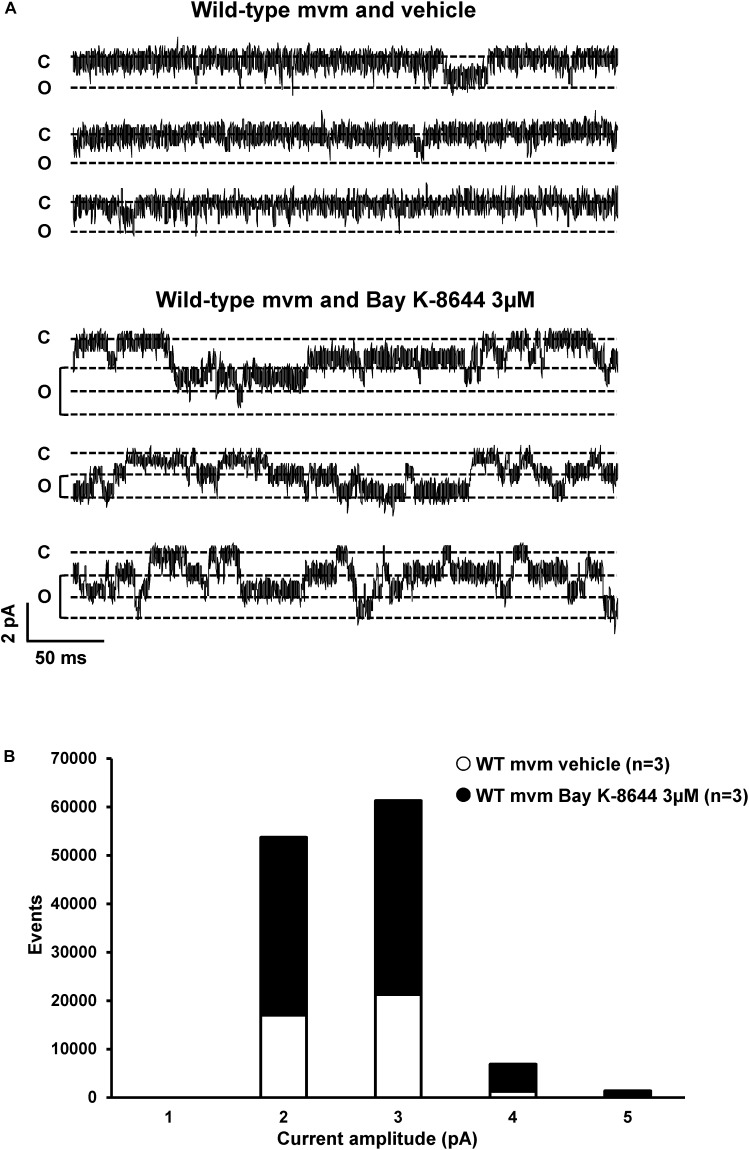
Calcium current recordings and cell-attached configuration. **(A)** Single currents recorded using cell-attached configuration on the lateral membrane of wild-type cardiomyocytes with only calcium inside the pipette. The calcium channel opener Bay K-8644 affects calcium currents, suggesting that this single current is a calcium current. **(B)** Quantification of events observed in panel **(A)** without (∘) or with (∙) Bay K-8644. The number of cells is indicated in parentheses.

Lastly, we assessed whether the cell-attached patch-clamp configuration allows the recording of T-tubular currents. In theory, the pipettes used in this study had an opening large enough (∼20 μm^2^) to catch the mouth of 3–5 T-tubules. We recorded I_Ca_ from lateral membrane on wild-type ventricular myocytes using an intrapipette solution with only barium as the charge carrier. Note that most of the cardiac voltage-gated calcium channels Ca_v_1.2 are located in T-tubules (Kawai et al., 1999). Contrary to the expected macroscopic I_Ca_, only single-channel current activities were observed, most likely arising from calcium channels (Figure 11A,B). To characterize these currents, 3 μM of the calcium channel opener Bay-K-8644 was added to the intrapipette solution. Bay-K-8644 significantly increased the number of events (vehicle: 39,677 events, *n* = 3; Bay-K-8644: 83,697 events, *n* = 3, *p* < 0.05; Figure 11A,B). Altogether, these results demonstrate that the cell-attached patch-clamp configuration on the lateral membrane does not permit to investigate ion channels activity at the T-tubules, since the size of the recorded I_Ca_ was much smaller than the one expected from T-tubules.

## Discussion

The present study shows that (1) a distinct population of Na_v_1.5 channels at the lateral membrane of mouse cardiomyocytes does not depend on either the PDZ-binding SIV motif or the α1-syntrophin protein; (2) the remaining I_Na_ at the lateral membrane of Na_v_1.5 ΔSIV myocytes is carried by sodium channels that are most likely not located in the T-tubules; (3) the identity of this sub-pool of sodium channels remaining at the lateral membrane is probably Na_v_1.5; and (4) the cell-attached patch-clamp configuration does not allow the investigation of ion channels expressed in the T-tubular system.

Although other Na_v_1.*x* channels (so-called neuronal isoforms) have been proposed to be expressed in the T-tubular system, recent transcriptomic analyses from freshly isolated murine ventricular myocytes suggests that only transcripts encoding Na_v_1.5 and Na_v_1.4 channels are significantly expressed (Chevalier et al., 2018). However, the presence of a Na_v_1.5 channel population in T-tubules of murine cardiomyocytes is still an open question. In ventricular cardiomyocytes, the punctate pattern observed in staining by Shy et al. and colleagues suggesting the presence of Na_v_1.5 channels inside the T-tubules could be due to either their presence at the Z-line level, a structure close to the T-tubules, or unspecific staining (Ziane et al., 2010; Shy et al., 2014). Cell-attached patch-clamp configuration on lateral membrane of wild-type and detubulated ventricular cardiomyocytes to record sodium currents has been proposed as an approach to decipher if sodium channels are expressed in T-tubules. Assuming that the T-tubular system accounts for an important percentage of the cell surface area, ∼30% decrease after detubulation of the cell capacitance measured in this study and in others suggest a poor detubulation procedure that may lead to a misinterpretation the results (Brette et al., 2002). Optical measurements have shown that a decrease of 32% of the cell capacitance, after formamide detubulation, corresponds *de facto* to 65% decrease of the cell surface area (Pasek et al., 2008). Based on these data, the results presented in Figure 10 clearly suggest that the cell-attached configuration does not allow the recording of the activity of sodium channels located in T-tubules. This conclusion is also supported by the recordings of unitary calcium conductances (as in Figure 11) that are most likely reflecting Ca_v_1.2 channel activity around the opening of T-tubule as reported by Lab et al. (2013).

The novel findings from the cardiac-specific α1-syntrophin knockdown mouse line suggest that the remaining I_Na_ at the lateral membrane is indeed independent of the interaction between α1-syntrophin with the Na_v_1.5 C-terminal SIV motif or, as Shy et al. (2014) and Matamoros et al. (2016) proposed, *via* the N-terminal SLA motif. Surprisingly, the decrease of the lateral membrane I_Na_ is somewhat less pronounced in α1-syntrophin knockdown than in ΔSIV cardiomyocytes suggesting that the SIV-motif may interact with other partners. However, one has to take into account that both mouse lines are on different genetic backgrounds. Therefore, caution should be taken before comparing these two set of data. Further experiments using a double ΔSIV-α1-syntrophin knockdown mouse model will have to be carried out to challenge this question. Such experiments would indubitably show whether the remaining I_Na_ at the lateral membrane is independent of both the SIV motif and α1-syntrophin.

It may seem surprising that the AP duration in cardiac-specific α1-syntrophin knockdown myocytes was not prolonged. It has been reported that mutations in the gene coding for α1-syntrophin lead to an increase of the late sodium current, and hence prolong the AP duration (Ueda et al., 2008; Cheng et al., 2009). This mechanism has been proposed to be caused by a decrease of interaction between mutant α1-syntrophin and the plasma membrane Ca^2+^ ATPase subtype 4b (PMCA4b) and scavenging nNOS. The concomitant upregulation of nNOS, and hyper-S-nitrosylation of Na_v_1.5 channel was proposed to underlie the increase of the late sodium current (Ueda et al., 2008; Cheng et al., 2009). Since in the α1-syntrophin knockdown model of this study the syntrophin protein is absent (Figure 1A, 2A), we speculate that the macro-molecular complex comprising Na_v_1.5, PMCA4b, nNOS, and α1-syntrophin is entirely dissociated. This may explain why no effect on the cardiac AP duration was observed.

The finding that only I_Na_ and not I_to_ and I_K1_ was altered in α1-syntrophin knockdown cardiomyocytes is at odds with the recent studies by Iwata et al. (2005) and Matamoros et al. (2016). This discrepancy may reflect fundamental differences when studying ion channel regulation using *in vitro* vs. *in vivo* models as already observed with the regulation of Na_v_1.5 by SAP97 (Petitprez et al., 2011; Milstein et al., 2012; Gillet et al., 2015; Matamoros et al., 2016). It appears unlikely that the remaining sodium channels at the lateral membrane interacts with other syntrophin isoforms since α1-syntrophin accounts for ∼90% of all syntrophin mRNA expressed in murine cardiac cells (Iwata et al., 2005; Chevalier et al., 2018). However, further experiments have to be performed using all-syntrophin isoform knockdown mouse strain to challenge this possibility. Moreover, although western blots and immunostaining experiments strongly suggest that α1-syntrophin expression is almost completely abolished, we cannot exclude the option that a very low amount of α1-syntrophin is still present in the knockdown model, which could contribute to a small portion of the remaining lateral membrane I_Na_.

The main limitations of the present study are primarily related to the methodological approaches. Future experiments could investigate the precise localization of these syntrophin-independent sub-pools of Na_v_1.5 channels at the lateral membrane using the scanning ion conductance microscopy (SICM) method. In addition, the different techniques used in this study were insufficient to unequivocally demonstrate whether Na_v_1.5 channels are present or absent inside T-tubules. Furthermore, when interpreting these data, we have to keep in mind that the compartmentalization of sodium channels in cardiomyocytes may be species-dependent. Using a similar approach as in the present study, Verkerk et al. (2007) showed that TTX-resistant voltage-gated sodium channels including Na_v_1.5 are absent at the lateral membrane of rabbit ventricular cardiomyocytes.

In summary, these results suggest the presence of a sub-pool of Na_v_1.5 channels at the lateral membrane of murine cardiomyocytes, outside the T-tubules, that is independent of α1-syntrophin and the SIV motif of Na_v_1.5. Future experiments will help to understand the functional roles of these distinct pools of sodium channels in specific membrane compartments of cardiomyocytes.

## Ethics Statement

All experiments involving animals were performed according to the Swiss Federal Animal Protection Law and had been approved by the Cantonal Veterinary Administration, Bern. This investigation conforms to the Guide for the Care and Use of Laboratory Animals, published by the US National Institutes of Health (NIH publication no. 85–23, revised 1996).

## Author Contributions

J-SR and HA conceived and designed the experiments. J-SR, HA, ME, LG, and SG collected, analyzed, and interpreted the data. SS and DS produced the alpha1-syntrophin transgenic mouse model. J-SR and HA drafted the manuscript and revised it critically for important intellectual content.

## Conflict of Interest Statement

SS and DS were employed by the company PolyGene AG, Rümlang, Switzerland. The remaining authors declare that the research was conducted in the absence of any commercial or financial relationships that could be construed as a potential conflict of interest.

## Supplementary Material

FIGURE S1 Generation of the *Snta1*-flox mouse model. **(A)** The diagram shows homologous recombination of the *Snta1* locus (Snta1-WT) with the targeting vector pSnta1-TV and the resulting targeted Snta1 alleles. Two loxP sites are inserted into the Snta1 locus, flanking a region of 0.7 kb that contains part of exon 1 of Snta1. One loxP site is inserted into the 5′ untranslated region (5′ UTR) of exon 1, whereas the second site is located in intron 1. Directly upstream of the 3′ loxP site, a neomycin resistance cassette (neo) is inserted, which is flanked by FRT-sites, the recognition sites for the Flp-recombinase (Snta1-neoflox allele). Upon breeding to Flp-deleter mice, the neomycin resistance cassette is deleted, and only a single FRT site is remaining in the *Snta1*-flox allele. The short (SA) and long homology regions (LA) are indicated as gray boxes. Positions of primers and sizes of products for genotyping PCR, as well as restriction sites and the probes for Southern blot analyses, are indicated. **(B)** Representative result of Southern blot analysis with genomic DNA from Snta1 wt/wt and Snta1 neoflox/wt (1C9) ES cells cut with PstI and probed using either a sequence upstream of exon 1 or downstream of exon 2 of Snta1. This resulted in a 14.3 kb signal for the wild-type allele with both probes and either a 4.7 kb signal for the 5′ probe and a 9.0 kb signal for the 3′ probe in the Snta1-neoflox allele. **(C)** PCR analysis of all three genotypes using the primer depicted in **A**. The wild-type (WT) allele resulted in a PCR product of 274 bp, whereas a 443 bp fragment was generated for the Snta1-flox allele (Flox).

FIGURE S2 Whole-cell biophysical properties of wild-type and *Snta1*-flox cardiomyocytes. **(A,B)** Activation **(A)** and steady-state inactivation curves **(B)** calculated from whole-cell recordings of sodium currents show no alteration of the biophysical properties between adult ventricular cardiomyocytes from wild-type (*Snta1* fl/fl^-^) and α1-syntrophin cardiac specific knockdown cardiomyocytes (*Snta1* fl/fl^+^). The number of cells is indicated in parentheses.

FIGURE S3 Cell-attached biophysical properties of wild-type and *Snta1*-flox cardiomyocytes. **(A,B)** Activation **(A)** and steady-state inactivation curves **(B)** calculated from cell-attached recordings of sodium currents show no alteration of the biophysical properties between adult ventricular cardiomyocytes from wild-type (*Snta1* fl/fl^-^) and α1-syntrophin cardiac specific knockdown cardiomyocytes (*Snta1* fl/fl^+^). The number of cells is indicated in parentheses.

FIGURE S4 Lateral sodium current measurements in wild-type and ΔSIV cardiomyocytes. **(A)** representative cell-attached recordings of sodium currents from the lateral membrane of wild-type and ΔSIV cardiomyocytes. **(B)** Current-voltage (I–V) curve showing a significant decrease in the sodium current recorded in ΔSIV cardiomyocytes (∙) compared to the control (∘) (^∗^*p* < 0.05). The number of cells is indicated in parentheses.

FIGURE S5 Cell-attached biophysical properties of wild-type and ΔSIV cardiomyocytes. **(A,B)** Activation **(A)** and steady-state inactivation curves **(B)** calculated from cell-attached recordings of sodium currents show no alteration of the biophysical properties between adult ventricular cardiomyocytes from wild-type and ΔSIV cardiomyocytes. The number of cells is indicated in parentheses.

FIGURE S6 Pharmacological block of Na_v_1.4 current in HEK-293 cells. **(A)** Representative cell-attached Na_v_1.4 currents recorded in transiently transfected HEK-293 cells. **(B)** I_Na_ reduction mediated by tetrodotoxin (TTX) after 3 min of application (^∗∗^*p* < 0.01). The number of cells is indicated in parentheses.
